# Action Observation for Neurorehabilitation in Apraxia

**DOI:** 10.3389/fneur.2019.00309

**Published:** 2019-04-03

**Authors:** Mariella Pazzaglia, Giulia Galli

**Affiliations:** ^1^Department of Psychology, University of Rome “La Sapienza”, Rome, Italy; ^2^IRCCS Fondazione Santa Lucia, Rome, Italy

**Keywords:** apraxia, action recognition, action execution, mirror activity, neurorehabilitation

## Abstract

Neurorehabilitation and brain stimulation studies of post-stroke patients suggest that action-observation effects can lead to rapid improvements in the recovery of motor functions and long-term motor cortical reorganization. Apraxia is a clinically important disorder characterized by marked impairment in representing and performing skillful movements [gestures], which limits many daily activities and impedes independent functioning. Recent clinical research has revealed errors of visuo-motor integration in patients with apraxia. This paper presents a rehabilitative perspective focusing on the possibility of action observation as a therapeutic treatment for patients with apraxia. This perspective also outlines impacts on neurorehabilitation and brain repair following the reinforcement of the perceptual-motor coupling. To date, interventions based primarily on action observation in apraxia have not been undertaken.

## Introduction

Apraxia encompasses a broad spectrum of higher-order purposeful movement disorders (1) and is most often associated with neurological damage to left-hemisphere (2). The accepted definition of apraxia includes deficits in performing, imitating, and recognizing skilled actions involved in the intentional movements, colloquially referred to as gestures (3). Pathological conditions such as apraxia result from an inability to evince the concept of specific actions (4) or to execute related motor programs (5). Classically, apraxia is diagnosed when a patient presents with an inability to execute gestures in response to verbal commands or imitate with different effectors (mouth, hand, or foot) (4), including movements involving the non-paretic limb ipsilateral to the lesion[s]. Although apraxia primarily affects motor activities, studies report that higher impairment levels may be related to visuo-motor integration (6). Recent evidence supports the notion that apraxia influences skilled acts in the environment, interferes with independent functioning, impedes daily activities, and affects the performance of routine self-care (7, 8); that is, persons may have difficulty brushing their teeth (9), eating (7), preparing food (10), and getting dressed (11). As a consequence, patients with apraxia can develop severe anxiety and reductions in the spontaneous use of social gestures (12), leading to isolation and depression (13) and consequent delays in returning to work (14).

Almost 50% of patients with left-hemispheric stroke (15) and ~35% of patients with Alzheimer's disease and corticobasal degeneration (16–18) develop apraxia that persists after illness onset and affects functional abilities. Research to aid in the development and optimization of apraxia neurorehabilitation is crucial. Several approaches for the treatment of apraxia deficits are currently in practice [for a review see (19, 20)], including verbal (21) or pictorial (22) facilitation and the use of physical cues based on repetitive behavioral-training programs with gesture-production exercises. The errorless completion method represents another recent approach (23). Autonomy in activities of daily living tends to be underestimated (24), and rehabilitation studies remain limited due to the nature of disturbances to automatic/voluntary dissociations (i.e., an ability to execute actions only in natural settings). To date, no rehabilitation treatment or therapeutic possibilities based primary on action observation has been studied in apraxia.

## The Value of Action Observation in Treating Apraxia

Language disorders among patients with apraxia who suffer from concomitant aphasia suggest that defects in gesture imitation, rather than gestures in response to verbal commands, are more sensitive indicators of apraxia (25). Goldenberg has proposed that imitation apraxia could be primarily considered a deficit of perceptual analysis (26). Evidence from several studies indicates that perceptual and motor codes are closely associated (27, 28) and that patients with apraxia may be defective both in performing motor acts and in the perceptual code necessary to represent the appropriate gesture. Sunderland and Sluman have shown, for example, that problems orienting a spoon in a bean-spooning task suggest an inability to remember the correct action and to judge the correctness of the perceived action (29).

Although apraxia is commonly considered a motor impairment, deficits in intact gestural perception are not uncommon, occurring in 33% of one sample (30). Such patients, who exhibit deficits in the execution of actions, also commit errors when judging between correctly and incorrectly performed acts (30–32), understanding the meaning of pantomimes (33, 34), discriminating among action-related sounds (35, 36), matching photographs of gestures (26), engaging visuo-motor temporal integration (6), and predicting incoming observed movements (37, 38).

Movement-execution effects in apraxia thus are not purely motor processes and visual representations of given actions may influence the actions' execution by visuo-motor transfer (39). The integrity of gesture representations has important implications for rehabilitation strategies (40). The spatial and temporal use of a body part for the planning of a tool-related action and the imitation of others' actions involve an inherent perceptual component, which can be disturbed following apraxia onset. As a result, modern assessments of apraxia include evaluations of gesture understanding (32, 41).

## Visual-Motor Strategies in the Rehabilitation of Patients With Limb Apraxia

The notion of common representations for both executed and observed actions is of considerable interest in the applied field of stroke neurorehabilitation (42, 43). Despite the use of state-of-the-art apraxia-evaluation batteries (44) to explore perceptual deficits in the understanding of actions in patients with apraxia, few studies have proposed new rehabilitation programs that include elements of both observation and execution of actions.

Smania et al.'s (45) clinical examinations of 43 left brain-damaged patients with apraxia revealed defective performances in gesture execution and imitation, as well as in the recognition and identification of transitive and intransitive gestures. For their study, approximately half of the patients received training in ecological action production and comprehension; the other half underwent conventional language rehabilitation for the same number of treatment hours. The training, which combined the observation and execution of observed actions, consisted of three progressive phases, each characterized by increasing degrees of difficulty, obtained by phased reductions of facilitation cues as performance improved. After ~30 sessions, therapists recorded significant improvements: approximately 50% improvement in the ADL scale and an average of 40% in the praxis test (22). When only considering apraxia patients with cortical lesions primarily in the fronto-parietal network, the improvement was even greater (45). No significant performance changes were observed in the outcome measures of control patients who did not undergo specific programs of gesture production/observation exercises. Interestingly, authors reported a significant improvement in gesture recognition performance after the apraxia treatment, and a correlation was found between gesture comprehension tests and the ADL questionnaire (ADL-gesture comprehension: *R* = 0.37, *p* = 0.034) (22). These results suggest that the positive effects of this rehabilitative approach in apraxia require parity in the treatment of both the motor and the perceptual aspects of action processing (45). Of note, beneficial effects persisted for at least 2 months and extended to the daily living activities even of untreated actions, helping patients attain functional independence from their caregivers (22).

Goldenberg and Hagmann (9) developed a particularly successful restorative method in which training comprised two different methods. The first aimed at helping patients to learn and correctly execute complete activities, with therapists providing different support at all clinical steps (e.g., by demonstrating gesture execution and asking patients to imitate them), and reducing the support only when patients were able to perform these steps on their own. The second aimed at directing patients' attention to the functional meaning of objects' individual features and details, critical for various actions. This two-step procedure ensured a double reinforcement of the action's perceptual-motor code: the first online within the simultaneity of the demonstration and the second off-line as a delayed imitation. The combination of these two methods led to significant improvements in trained ADL, but virtually no generalization of training effects was observed between trained and non-trained activities. The therapy's success was preserved among those patients who performed the activities at home but not among those who did not. In a subsequent study (46), the authors developed a slightly different variant to previous approaches in which patients carried out entire activities with a minimum of errors. In this approach, the functional commonalities between different objects were emphasized by providing verbal instructions and visual and gestural support. Effects of these treatments lasted up to 3 months after the treatment ended.

Compensatory treatment indicate that the patients showed large improvements in ADL functioning after rehabilitative programs aiming at teaching visual strategies to overcome the apraxic impairments during execution of everyday activities (47). Patients were taught strategies to compensate internally (e.g., self-verbalization or imagination) or externally (e.g., observation of pictorial cues) the distinct phases of a complex action, while performing the daily activities (47–50).

All described interventions included elements of visuo-motor integration and seemed to indicate that motor and visual relearning in these patients was inextricably intertwined (see Table 1).

**Table 1:** Apraxia intervention studies.

**References**	**Number of participants**		**Treatment duration**	**Type of action**	**Control**	**Intervention**	**Perceptual aspects of training**	**Improvements in experimental group**	**No effect**
	**Experimental group**	**Control group**							
van Heugten et al. (47)	33		30 min for 12 weeks	Everyday activities		Strategy training	Observation of picture sequences Imagination	ADL Barthel Index Apraxia Test Motor functioning	
Goldenberg and Hagmann (9)	15		5 weeks	Three activities from the domains eating, dressing, and grooming		Direct training of the activity: errorless completion of the activity	The patients perform action immediately after observing the therapist's demonstration	10 patients improved on all three trained activities	6 months later, improvement is not maintained without practice
Smania et al. (45)	6	7	35 sessions, three per week	Transitive action Intransitive action Imitation	Aphasia therapy	Gesture recognition Gesture execution	Observation of picture (context, object) Gesture recognition Imitation	Apraxia Test Gesture recognition	Verbal comprehension Oral apraxia
Donkervoort et al. (48)	42	48	8 weeks	Everyday activities	Occupational therapy	Strategy training	Observation of picture sequences Imagination	ADL Barthel Index	Apraxia Test ADL untrained
Goldenberg et al. (46)	6		4 weeks	Four everyday activities		Explorative training vs. Direct training of the activity	The patients perform action immediately after observing the therapist's demonstration	Direct training of activity reduced errors and amount of assistance	Exploration training had no effect on performance
Smania et al. (22)	18	15	30 sessions, three per week	Transitive action Intransitive action Imitation	Aphasia therapy	Gesture recognition Gesture execution	Observation of picture (context, object) Gesture recognition Imitation	Apraxia Test Gesture recognition ADL	Verbal comprehension Oral apraxia
Geusgens et al. (50)	56	57	25 sessions, 8 weeks	Action of daily living	Occupational therapy	Strategy training	Observation of picture sequences	ADL untrained	
Geusgens et al. (49)	29		25 sessions, 8 weeks	Action of daily living		Strategy training	Observation of picture sequences	Apraxia Test ADL trained ADL untrained Barthel Index	Functional Motor Test
Bolognini et al. (51)	6	6	3 sessions, 10 min	Limb gesture imitation	Sham stimulation	Anodal tDCS on the left parietal cortex	Imitation (observation + execution)	Imitation execution	tDCS on the motor cortex

**Figure 1:**
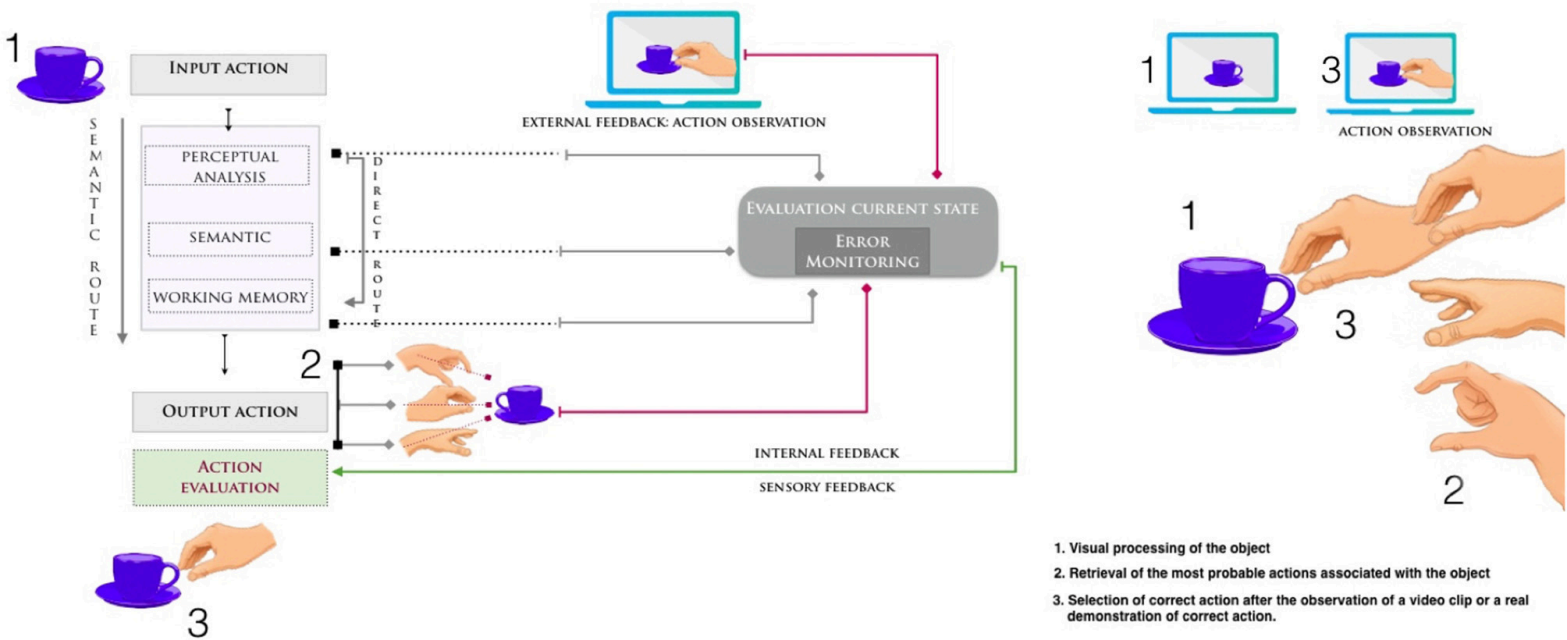
Hypothetical model for performing and recognizing a transitive action [adapted from (54) and (55)]. Failures in performing or recognizing gestures may occur because of damage at any stage in the directional flow between perceiving (input) and performing (output) the action. The observation of a video clip or a real demonstration of action can have a positive effect on the selection and retrieval of the correct movement. In figure the example of grasping a cup of coffee. After the correct visual identification of the object as a cup, patients with apraxia have a difficult retrieval of the correct action associated with that object. When an incorrect movement is performed, a discrepancy occurs between the (correct) action observed on the model and the perception of own (incorrect) performed gesture. Combining motor training and action observation may enhance the relearning of daily actions and strengthen the visuo-motor coupling.

Perceptual approach has been successfully applied to a different rehabilitative intervention showing how action observation has a positive effect on the performance of a specific motor skill [for a review see (41, 52, 53)]. Patients watch a specific motor act presented in a video clip or in a real demonstration, and simultaneously (or thereafter) performed the same action. A match (or mismatch) between visual signals and the gesture performed drive re-learning about how the limb should move in order to perform the motor act accurately (see Figure 1 for a hypothetical model on apraxia). Correctly reproducing temporal (56, 57), spatial (58), and body coding (59) helps characterize movements, facilitate the motor patterns that patients have to execute, and stimulate a rapid online correction of movement (58, 60, 61). Observation combined with physical practice in a congruent mode leads to increased motor cortex excitability, and synaptic and cortical map plasticity strengthens the memory trace of the motor act (62). Differently, rehabilitative training based on physical practice alone (300–1,000 daily repetitions) elicits only minimal neural reorganization (63). This combined visual-motor therapy has been shown to improve motor performance in patients that suffered a chronic stroke (64–86), patients with Parkinson's disease (87–92), children with cerebral palsy (93–97) and elderly individuals with reduced cognitive abilities (98). Electrophysiological studies have also reported positive effects of action observation on the recovery of motor functions after acute and chronic stroke (71, 99). This non-invasive, inexpensive, user-friendly approach works more quickly on biological effectors (mouth, limbs, and trunk), promoting better and faster recovery.

## A Neural Substrate for Action Observation and Execution in Apraxia Rehabilitation

The inextricable link between action perception and execution was first posited in the ideomotor theory, which has been validated through delineation of the brain network, known as the mirror neuron system (MNS). Inspired by single-cell (“mirror neuron”) recordings in monkeys (100, 101), many neuroimaging and neurophysiological studies have suggested that the adult human brain is equipped with neural systems and mechanisms that represent both the visual perception and execution of actions in a common format (102). Action deficits among the patients with apraxia may be described at multiple levels. While these levels partially overlap, four levels of hierarchical modeling at which an MNS mechanism can support an observed action (42, 103) are as follows:

- *kinematic*: Patients with apraxia frequently present with abnormalities in kinematic movements in the form of motor patterns that are slower, shorter, and less vertical than those of individuals without apraxia (104);
- *motor*: Limb apraxia interferes with the selection and control of the hand-muscle activity (105). Moreover, it interferes with the formation of appropriate hand configurations for using objects (106);
- *goal*: Understanding the immediate purpose of an action is impeded; for example, patients with apraxia are impaired access to mental representation of tool use (33);
- *intention*: Patients present with an altered ability to monitor the early planning phases of their own actions (107).

The cortical areas have been shown to contain mirror neurons that are often described as a part of an integrated sensorimotor information system underpinned by neural activity in the frontal (103), parietal (108), and superior temporal sulcus areas. This system is called the action observation network (AON) (109). In humans, these cortical regions mediate the observation of actions that form a part of the observer's motor repertoire (41). They also contribute to the imitation (110) and comprehension (111) of these movements, and are involved in skill acquisition (112). Lesion symptom mapping studies have reported gestural deficits in patients with apraxia, which are most frequently apparent following lesions in the inferior frontal lobe (30, 113–116), and in supramarginal and angular gyrus (37, 113, 115, 117) of the left hemisphere. However, apraxia has also been observed in patients with damage in posterior middle temporal lobe, anterior temporal lobe (37, 113, 115, 117), occipital, and subcortical regions (6, 118, 119). Despite the damaged neural substrate was not constant across all the studies, it includes the areas that are considered crucial for the AON. Undoubtedly, the mirror neurons just provide a part of the complex information for achieving action comprehension while action recognition and production occur simultaneously by accessing the same neural representations. However, as posited by the influential cognitive neuropsychological models of apraxia (120, 121) and demonstrated by various clinical studies (121–124), the range of possible dissociations between action execution and action understanding that can occur in patients with apraxia is quite multifaceted and cannot be explained by a mere action mirroring mechanism nor by a single lesion locus. Impairments in the visual recognition of action paralleled deficits in performing these actions could depend on both common and distinct neural localization, most of which could be external to mirror regions. Failures in imitating or in recognizing gestures may occur because of damage at any level in the process between perceiving (input lexicon) and performing (output lexicon) an action (120, 121). Indeed, some apraxic patients show deficits in the recognition/discrimination of the gestures, some do not [for a review (125)]. Theoretical and empirical studies suggest that complementary routes to action understanding taking place on the dorso-dorsal and ventro-dorsal stream (126, 127). Lesion in ventral-dorsal stream may impede the top-down activation of motor engrams. It may produce disturbances in the on-line selection and integration of distinctive and relevant motor acts that ensure a high recognizability of the gesture (117). This can be responsible for the disordered motor planning, imitation, and motor-memory recall of gesture movements found in patients with apraxia (126, 127). As has been briefly shown, many questions remain, and there may be more than one mechanism leading to apraxia disturb. Given the complexity of the impairment and the separate neural substrates that are typically affected in apraxia, treatments related to action observation to support action execution or relearning of gestures of daily living, can be planned.

## Neurorehabilitation and Brain Repair After Apraxia

The behavioral success of rehabilitation methods based on the principle of action observation should promote reorganization by adaptive plasticity at the neural level (128, 129). Functional reorganization clearly depends on the residual neural integrity of efferent (motor) and afferent (sensory) information, which leads to improved treatment outcomes among some apraxia patients but not for others. In this perspective, we considered three possible sources of informational content for how neurorehabilitation and brain repair after apraxia works: injury site, elapsed time after apraxia onset, and lesion size.

The first factor to consider is *the location of the infarct*, which can ultimately determine the outcome of rehabilitation treatment. Whereas, lesions of the frontal and parietal cortices in the left hemisphere have been shown to primarily disrupt gesture production in patients with apraxia (2), no clear correlation has been found between lesion location and impairment in visual gesture representation. Apraxic patients with cortical lesions—but not those with subcortical lesions—cannot comprehend the meaning of gestures (130). In rare cases, a lesion in the left occipito-temporal cortex may also critically hamper the ability to recognize gestures in patients with apraxia (120, 131). Patients with parietal lesions have also been reported to exhibit significant impairments in executing gestures but only slight impairments in understanding those performed by others (132). The neural specificity of this disturbed typology may explain why certain patients with apraxia are able to comprehend the meaning of gestures despite being unable to perform them themselves. Accordingly, single-case and group studies report dissociations between action execution and representation and the underpinning damaged neural substrate (121–124). Efficiency and speed of the therapeutic means of action observation depend partly on the different roles that intact and damaged brain regions play in both action production and recognition (125, 133). Neural damage to a functional system can be partial, and studies in monkeys seem to suggest that the frontal and parietal cortices are neurally equipped for such divisions of labor (134).

Several studies have documented that neurorehabilitation techniques involving observation strategies among brain-damaged patients induce long-lasting neural changes in the motor cortex, potentiating activity in the affected areas. In brain-damaged patients, TMS studies have found direct evidence of increased motor-cortex excitability (84), and synaptic and cortical map plasticity have been documented using fMRI (75). TMS studies have also indicated that action observation alone is able to drive reorganization in the primary motor cortex, strengthening the motor memory of observed actions among young (135) and elderly subjects (mean ages: 34 and 65 years, respectively) (98) and among chronically brain-damaged patients (84). Additionally, a study reported positive effects on gesture imitation of anodal transcranial direct current stimulation (tDCS) on the left parietal compared to sham tDCS, supporting the view that apraxia disorders in Parkinson (136) and in brain left damaged patients (51) can be improved by stimulating distinct structures.

A second factor to consider is the *temporal stage of the illness*. The neural substrates of action production and comprehension could be associated with different physiological mechanisms at different temporal stages of apraxia. Frontal and parietal areas may become temporarily inactive because of cerebral edema and intracranial hypertension, hemodynamic signs of ischemic penumbra, or local inflammatory effects in acute but not chronic stages of apraxia (137). Different studies report that during early periods (including an acute four-week, post-onset phase), impaired gesture recognition may be associated with left frontal–lobe and basal-ganglia lesions (138), whereas in the chronic stages of the illness, these deficits can be associated with left-parietal lesions (32, 37).

In practice, transitory effects such as the inability to mimic actions from visual cues are often observed in apraxia's early stages. If so, an observation intervention in early therapy may be inefficacy.

During later apraxia stages, a close overlap of the networks underlying observation and execution, as indicated by advanced neuroimaging and the lesion locations studies in patients, are helpful in identifying patient in which observative approach is potentially useful. Observation therapy associated with adaptive neurophysiological and neurometabolic changes can be conducted even several years after stroke onset. A session of 4 weeks of active, 18 days-cycle visual/motor training has been found to significantly enhance motor function, with increases in the activity of specific motor areas that possess mirror properties (75). Massed, high-frequency rehabilitative training (300–1,000 daily repetitions) is needed to elicit minimal neural reorganization (63). These increases in cortical activity during both action observation and execution also tend to be present in the hemispheres (139, 140) close to and far from the lesion site.

A third possible factor to consider is that the failure to link perceptual and motor representations in apraxia treatment may be *an effect of infarct size*; larger lesions are more likely to include front parietal injury and may not benefit from observation treatment. Indeed, improvements in imitation (reproduction off-line of the observed gesture) in patients with apraxia are influenced by the size of the parietal lesion (51): the larger the left parietal damage, the smaller the tDCS treatment-related improvement. When a functional system is completely damaged, however, recovery is achieved largely by process of substitution and may depend on the implicit engagement of neural systems to take over the functions of the damaged areas (141).

Whereas, some systems may constitute the sites of gesture performance, others may reduce the impact of deficits (142) by stimulating coupled visual knowledge mechanisms (98). The integrity of both the frontal and parietal cortices might be crucial for re-learning as a result of motor mirroring. Nonetheless, non-injured cortical areas could also trigger additional, independent internal mechanisms that support but are not necessary for guiding the motor system to match vision with motor routines (143, 144). Studies on the neural representations of motor skills based on observations of the motor cortex of macaque monkeys (145) and humans (146) provide empirical support for such an alternative system. These studies suggest that congruent activity during action execution/observation occurs even outside the canonical “mirror area,” representing a potential general property of the motor system. Targeting interventions on the basis of specific brain structures intact and damaged that could mediate the effects of training is an important future challenge in cognitive neurorehabilitation.

## Conclusion

While research on the relationship between observed and executed actions in apraxia neurorehabilitation has a short history, it has already provided insights about the positive effect of a visual-motor training. The observation of actions through a process of visual retrieval may help in the selection of the most probable action, providing a powerful tool for overcoming intentional motor-gestural difficulties (55). Moreover, tailored interventions based on individual's ability to acquire new (or relearn old) motor-memory traces through multisensory [i.e., auditory (35, 147), olfactory (148, 149), and tactile (150–155)] feedback may be the most promising approach for a normal temporal integration action (156, 157). Multisensory stimulation can activate multiple cortical brain structures, inducing cortical reorganization and modulating motor cortical excitability for the stimulated afferents (158, 159). Results are encouraging, but it is important to emphasize that this hypothesis does not imply that all deficits in apraxia can be treated by action observation therapy. Rather, we believe that action observation might be a therapeutic option for improving praxis function among certain specific typologies of patients.

## Author Contributions

MP: study concept and design, manuscript development, and writing. GG: contributed to the writing of the manuscript.

### Conflict of Interest Statement

The authors declare that the research was conducted in the absence of any commercial or financial relationships that could be construed as a potential conflict of interest.
